# Evaluation of the CIB1R peptide derived from the cytoplasmic domain of neprilysin on cell migration in an *in vitro* model of lung cancer

**DOI:** 10.3389/fonc.2026.1825515

**Published:** 2026-06-23

**Authors:** Carlos Alejandro Martínez-Armenta, Horacio Almanza-Reyes, Leslie Patrón-Romero, Adriana Sampayo-Reyes, Juan M. Alcocer-González, Reyes Tamez-Guerra, Cristina Rodríguez-Padilla, Humberto Antonio Salazar-Sesatty, Omar Zardain-Medlich-Ducoulombier, Francisco González-Salazar, Javier Vargas-Villarreal

**Affiliations:** 1 Laboratory of Immunology and Virology, Faculty of Biological Sciences, Autonomous University of Nuevo León, San Nicolás de los Garza, Nuevo León, Mexico; 2 Faculty of Medicine and Psychology, Autonomous University of Baja California., Tijuana, Baja California, Mexico; 3 Department of Basic Sciences, Vice-Rectorate of Health Sciences, University of Monterrey, San Pedro Garza García, Nuevo León, Mexico; 4 Department of Molecular Biology, Northeast Biomedical Research Center, Mexican Institute of Social Security, Monterrey, Nuevo León, Mexico

**Keywords:** A549 cells, CIB1R peptide, lung cancer, metastasis, migration, therapeutic agent

## Abstract

**Purpose:**

Lung cancer is characterized by high metastatic potential and poor clinical outcomes, underscoring the need for novel approaches to investigate mechanisms regulating tumor cell migration and invasion. The CIB1R peptide, derived from the cytoplasmic domain of neprilysin (NEP), has been proposed as a potential modulator of these processes. This study aimed to evaluate the effects of CIB1R and related peptides on cellular behaviors associated with metastasis in an *in vitro* model of non-small cell lung cancer.

**Materials and methods:**

Peptides were prepared and administered under serum-free conditions. Cell viability was assessed using the Alamar Blue assay. Migration and invasion were evaluated using Transwell (Boyden chamber) assays, while cell adhesion was quantified on Matrigel-coated surfaces. Peptide-associated fluorescence was analyzed using flow cytometry and confocal microscopy with FITC-labeled peptides. Membrane integrity was assessed by propidium iodide exclusion. Statistical analyses were performed using one-way ANOVA, followed by the Tukey *post hoc* test. A p-value < 0.05 was considered statistically significant.

**Results:**

CIB1R reduced cell migration and invasion in A549 cells in a dose-dependent manner without significantly affecting cell viability over 24 hours. However, similar inhibitory effects were observed with structurally related peptides, including a scrambled control sequence, indicating that the observed activity is not strictly sequence-specific. All peptides demonstrated strong cell-associated fluorescence signals, although the experimental design did not allow definitive discrimination between membrane-associated and intracellular localization. No significant increase in membrane permeability was detected under the conditions tested.

**Conclusions:**

These findings provide initial functional evidence that short cationic peptides derived from, or related to, the cytoplasmic domain of NEP can modulate cellular behaviors associated with tumor progression *in vitro*. The observed effects appear to be influenced by shared physicochemical properties rather than strictly sequence-specific mechanisms. Further studies are required to clarify the molecular basis of these effects and their relevance under physiological conditions.

## Introduction

Cancer constitutes one of the leading causes of mortality worldwide. The number of deaths attributable to this disease is projected to continue increasing, with an estimated 11.5 million deaths by the year 2030 (1). Population aging significantly contributes to this trend, rendering cancer a major public health concern worldwide. Among its various forms, lung cancer (LC) is the leading cause of cancer-related deaths globally (2). Within its predominant histological subtypes, non-small cell lung cancer (NSCLC) accounts for approximately 76% of cases, while small cell lung cancer (SCLC) comprises 13% (3). NSCLC is further classified into three major subtypes: squamous cell carcinoma (SCC), adenocarcinoma (ADC), and large cell carcinoma (4). Despite advancements in therapeutic interventions and the standardization of clinical trials to enhance patient outcomes, the median survival of NSCLC patients remains less than one year following diagnosis (5). As the disease progresses, LC can affect various extrapulmonary structures, including serous membranes such as the pleura and pericardium, resulting in fluid accumulation within these cavities. These effusions may contain tumor-derived biomarkers, the quantification of which provides valuable information for determining the origin of the fluid accumulation, whether in the context of pleural effusion, pericardial effusion, or malignant ascites (6). Malignant ascites is characterized by the presence of tumor cells in the peritoneal fluid of patients with advanced metastatic disease. Its pathophysiology involves increased fluid filtration, neovascularization, and lymphatic obstruction, serving as a marker of poor prognosis (7). Approximately 50% of patients with advanced malignancies develop malignant pleural effusion and ascites during disease progression (8).

Previous research by the Palacios-Corona R. team investigated the immunosuppressive properties of protein fractions <10 kDa derived from ascitic fluid samples of patients with diverse primary neoplasms. Nitric oxide (NO) production was measured in peripheral blood mononuclear cells (PBMCs) stimulated with these fractions, lipopolysaccharides, N-Methyl-L-Arginine (a commercial NO production inhibitor), and benign ascitic fluid. Results showed that samples of malignant ascitic fluid significantly decreased NO concentrations compared to benign controls. Subsequently, liquid chromatography coupled with mass spectrometry (LC-ESI-MS/MS) was employed to sequence the <10 kDa fractions, thereby identifying peptide sequences of interest and their corresponding alignment proteins (9). Among these sequences, sequence 17 (PKPKKKQ) stands out, as it aligns with the neprilysin protein, which has been described as a key modulator of cell migration and proliferation in various types of neoplasms (10). Furthermore, the CIB1R sequence aligns with the cytoplasmic domain of neprilysin, which interacts with PTEN and phosphorylated Lyn to regulate cellular migration. Nonetheless, in NSCLC, neprilysin expression is diminished, thereby enhancing the tumor’s invasive potential (11). In this study, we assessed the impact of the CIB1R peptide on cellular migration and proliferation within an *in vitro* NSCLC model. These findings may contribute to the development of novel therapeutic strategies to counteract tumor growth, angiogenesis, and metastasis.

## Materials and methods

### Peptide resuspension

Four peptide sequences were acquired from the commercial supplier Bio-Basic Inc. (Markham, Ontario, Canada; RRID: SCR_012675): CIB1R (H_3_N^+^–PKPKKKQ–COO^-^), the working peptide; PS (H_3_N^+^–KQKPKKP–COO^-^), a scrambled version of the working peptide; CTT, a control peptide known to inhibit cell migration and invasion (12); and PC (H_3_N^+^–GKSESQMDITDINTPKPKKKQRWTPLE–COO^-^), representing the cytoplasmic domain of the neprilysin protein. Each sequence was synthesized with a purity >95%, as verified by high-performance liquid chromatography–tandem mass spectrometry (HPLC-MS/MS) conducted by the supplier. Lyophilized peptides were stored at –20 °C within a desiccator containing silica gel until use, in accordance with the manufacturer’s guidelines. For experimental purposes, the entire lyophilized content of each peptide was reconstituted in Milli-Q water (Merck Millipore, Darmstadt, Germany) to prepare stock solutions at 9 mg/mL, which were subsequently aliquoted and stored at –20 °C. Working dilutions were prepared in serum-free Dulbecco’s Modified Eagle Medium (DMEM, Thermo Fisher Scientific, Waltham, MA, USA) at the molar concentrations required for each assay and kept at 4 °C for 10 minutes before use.

### Cell culture

The human lung adenocarcinoma cell line A549 (ATCC^®^ CCL-185™, RRID: CVCL_0023) was used as the study model. The cells were originally acquired from the American Type Culture Collection (ATCC, Manassas, VA, USA) and kindly provided by Dr. Cristina Rodríguez Padilla, Head of the Immunology and Virology Laboratory at the Faculty of Biological Sciences of the Autonomous University of Nuevo Leon (UANL, Monterrey, Mexico). Cells were cultured in Dulbecco’s Modified Eagle Medium (DMEM; Gibco, Thermo Fisher Scientific, Waltham, MA, USA) supplemented with 10% heat-inactivated fetal bovine serum (FBS; Gibco, Thermo Fisher Scientific, USA), 100 U/mL penicillin, and 100 μg/mL streptomycin (Thermo Fisher Scientific, USA). Cultures were grown in 25 cm² tissue culture flasks (Corning Inc., Corning, NY, USA) under a humidified atmosphere containing 5% CO_2_ at 37 °C. Subculturing was performed when cells reached 70–80% confluence.

### Alamar Blue assay

Cells were seeded in 96-well flat-bottom plates (Corning Inc., Corning, NY, USA) at a density of 6 × 10³ cells per well in complete medium and incubated at 37 °C in a humidified atmosphere containing 5% CO_2_ for 24 hours to allow cell adhesion. Following incubation, the medium was replaced with fresh complete medium containing the indicated peptides at final concentrations of 800, 400, 200, 100, 50, and 25 μM, adjusted to a final volume of 0.1 mL per well. The plates were incubated for an additional 24 hours under identical conditions. After treatment removal, 100 μL of complete culture medium supplemented with 10 μL of Alamar Blue reagent (Thermo Fisher Scientific, Waltham, MA, USA; DAL1100) was added to each well, and the plates were further incubated for 2.5 hours at 37 °C in a 5% CO_2_ atmosphere. Cell viability was quantified by measuring absorbance at 570 nm using a BioTek Synergy HT microplate reader (Agilent, Santa Clara, CA, USA; RRID: SCR_020536). The positive control consisted of cells incubated in supplemented DMEM, whereas 0.1% Triton X-100 (Sigma-Aldrich, St. Louis, MO, USA) was employed as the negative control. The mean absorbance of the positive control was set to 100% cell viability. All experimental procedures were conducted in triplicate and independently replicated at least twice.

### In vitro analysis of the effect on cell migration

Cell migration was evaluated using Boyden chambers (Transwell cell culture inserts; Corning Inc., Corning, NY, USA; Cat. No. 3422) placed within 24-well plates. Each insert was equipped with a polycarbonate membrane featuring 8 μm pores, acting as a permeable support for cell migration assays. A549 cells were grown in 25 cm² flasks until reaching 80–90% confluence. Subsequently, the medium was substituted with serum-free DMEM (Gibco, Thermo Fisher Scientific, Waltham, MA, USA), and cells were incubated for 8 hours to achieve synchronization. The cells were then harvested via trypsinization, and the reaction was quenched by resuspending the cells in DMEM supplemented with 50 μg/μL bovine serum albumin (BSA; Sigma-Aldrich, St. Louis, MO, USA). A cell suspension of 2.5 × 10^5^ cells/mL was prepared in serum-free DMEM, and 100 μL of this suspension was allocated to the upper chamber of each insert along with the respective peptide treatments: CIB1R (800, 500, 200, and 100 μM), PC and PS (306 μM each), and CTT (500 μM). The lower chamber was filled with 0.65 mL of DMEM supplemented with 10% FBS (Gibco, Thermo Fisher Scientific, USA) to act as a chemoattractant. Following a 24-hour incubation at 37 °C in a humidified 5% CO_2_ atmosphere, non-migrated cells on the upper surface of the membrane were removed using a sterile cotton swab. Cells that had migrated to the lower surface were fixed in 4% paraformaldehyde (Sigma-Aldrich, USA) for 15 min, rinsed twice with PBS, and stained with DAPI (Thermo Fisher Scientific, USA). Imaging was performed using a Leica TCS SP8 AOBS DMI6000 multiphoton confocal microscope (Leica Microsystems, Wetzlar, Germany; RRID: SCR_027226). For each insert, five microscopic fields were randomly selected to quantify the number of migrated cells. Migration results were expressed as the percentage of cells migrated relative to the control, which was set to 100%. Each experiment was conducted in triplicate and independently replicated at least twice.

### Cell invasion assay

Cell invasion was assessed using Transwell cell culture inserts (8 μm pore size; Corning Inc., Corning, NY, USA; Cat. No. 3422) placed in 24-well plates. The upper surface of each membrane was coated with 70 μL of Matrigel^®^ Basement Membrane Matrix (0.6 mg/mL; Corning Inc., Cat. No. 356234) and incubated for 2 hours at 37 °C to allow polymerization. A suspension of 2.5 × 10^5^ A549 cells/mL was prepared in serum-free DMEM (Gibco, Thermo Fisher Scientific, Waltham, MA, USA) after cells were harvested through trypsinization from cultures that had been incubated for 8 hours in serum-free medium. Trypsin activity was inactivated by resuspending cells in DMEM containing 50 μg/μL bovine serum albumin (BSA; Sigma-Aldrich, St. Louis, MO, USA). Subsequently, 100 μL of the cell suspension was seeded in the upper compartment of each insert alongside the peptide under evaluation at a final concentration of 306 μM. The lower chamber was filled with 0.65 mL of DMEM supplemented with 10% FBS (Gibco, Thermo Fisher Scientific, USA), serving as a chemoattractant. Following 24 hours of incubation at 37 °C in a humidified 5% CO_2_ atmosphere, non-invading cells remaining on the upper surface of the membrane were removed using a sterile cotton swab. Invaded cells adhering to the lower surface of the membrane were fixed with 4% paraformaldehyde (Sigma-Aldrich, USA) for 15 minutes, rinsed twice with PBS, and stained with DAPI (Thermo Fisher Scientific, USA). Imaging was conducted using a Leica TCS SP8 AOBS DMI6000 multiphoton confocal microscope (Leica Microsystems, Wetzlar, Germany; RRID: SCR_027226). Five randomly chosen fields per insert were selected for quantification. Invasion was expressed as the percentage of invaded cells relative to the untreated control, arbitrarily set at 100%. All experiments were conducted in triplicate and independently replicated at least twice.

### Inhibition of cell adhesion

Cell adhesion was assessed using 96-well flat-bottom plates (Corning Inc., Corning, NY, USA) coated with 70 μL of Matrigel^®^ Basement Membrane Matrix (0.6 mg/mL; Corning Inc., Cat. No. 356234) and incubated at 37 °C for 2 hours to allow polymerization. A suspension of 2.5 × 10^5^ A549 cells/mL was prepared in complete medium, and 100 μL of this suspension was added to each well along with the peptide under assessment (306 μM). The plates were then incubated for 1 hour at 37 °C in a humidified atmosphere with 5% CO_2_. Following incubation, the treatment was removed, and the wells were carefully washed three times with sterile PBS (Gibco, Thermo Fisher Scientific, Waltham, MA, USA) to remove non-adherent cells. Subsequently, 100 μL of complete culture medium supplemented with 10 μL of Alamar Blue reagent (Thermo Fisher Scientific, USA; DAL1100) was added to each well, and plates were incubated for an additional 2.5 hours at 37 °C under 5% CO_2_. Absorbance was measured at 570 nm using a BioTek Synergy HT microplate reader (Agilent Technologies, Santa Clara, CA, USA; RRID: SCR_020536). Complete medium served as the positive control (100% adhesion), and the results were expressed relative to this value. Each experiment was conducted in triplicate and independently replicated at least twice.

### Uptake analysis via flow cytometry

Cellular uptake of FITC-labeled peptides was assessed through flow cytometry. A549 cells were seeded at a density of 3 × 10^4^ cells per well in 24-well plates (Corning Inc., Corning, NY, USA) and incubated at 37 °C within a humidified atmosphere containing 5% CO_2_ for 24 hours. Subsequently, cells were treated with the IC_50_ concentration (306 μM) of each FITC-labeled peptide (CIB1R, PS, and PC) and incubated for different time intervals (1, 3, 6, and 24 hours). Following peptide treatment, cells were washed twice with sterile PBS (Gibco, Thermo Fisher Scientific, Waltham, MA, USA) to remove unbound peptides. Cells were harvested through trypsinization, resuspended in PBS, and immediately analyzed using a BD Accuri™ C6 Plus Flow Cytometer (BD Biosciences, Franklin Lakes, NJ, USA; RRID: SCR_019591). Data collection encompassed at least 10,000 events per sample, with fluorescence measurements taken in the FITC channel (excitation 488 nm, emission 533/30 nm). Data were subsequently processed and analyzed employing FCS Express Flow Cytometry Analysis Software (*De Novo* Software, Pasadena, CA, USA; RRID: SCR_016431). All experimental procedures were conducted in triplicate and independently replicated at least twice.

### Fluorometric analysis via confocal microscopy

Cellular uptake of FITC-labeled peptides was further assessed through confocal microscopy. A549 cells were seeded at a density of 3 × 10^4^ cells per well in 24-well plates (Corning Inc., Corning, NY, USA) containing sterile porous glass coverslips, and incubated for 24 hours at 37 °C in a humidified atmosphere with 5% CO_2_. Subsequently, the cells were treated with the IC_50_ concentration (306 μM) of FITC-labeled peptides (CIB1R, PS, and PC) and incubated for an additional 6 hours under identical conditions. Post-incubation, the wells were thoroughly washed with sterile PBS (Gibco, Thermo Fisher Scientific, Waltham, MA, USA) to remove any unbound peptides. The cells were then counterstained with DAPI (Thermo Fisher Scientific, USA) for nuclear visualization, mounted on glass slides with antifade mounting medium (Thermo Fisher Scientific, USA), and protected from light until analysis. Imaging was conducted using a Leica TCS SP8 AOBS DMI6000 multiphoton confocal microscope (Leica Microsystems, Wetzlar, Germany; RRID: SCR_027226) with excitation/emission configurations suitable for FITC and DAPI detection. Representative fields were captured from each sample, and peptide internalization was qualitatively evaluated. All experiments were conducted in triplicate and independently replicated at least twice, with imaging carried out within 10 minutes of sample preparation to minimize fluorophore quenching.

### Membrane permeability assay (PI uptake)

Membrane integrity was evaluated through the assessment of propidium iodide (PI) uptake via flow cytometry. A549 cells were seeded at a density of 3 × 10^4^ cells per well in 24-well plates (Corning Inc., Corning, NY, USA) and incubated for 24 hours at 37 °C in a humidified atmosphere containing 5% CO_2_. Subsequently, the cells were exposed to the IC_50_ concentration (306 μM) of the peptide under investigation for 24 hours. Following treatment, the cells were washed with sterile PBS (Gibco, Thermo Fisher Scientific, Waltham, MA, USA), harvested by trypsinization, and neutralized with complete medium. The resulting suspensions were centrifuged at 300 × g for 5 minutes, washed once in PBS, and resuspended in 0.4 mL of staining solution containing 50 μg/mL propidium iodide (PI; Molecular Probes, Thermo Fisher Scientific, USA; Cat. No. P3566). The cells were subsequently incubated in the dark and at room temperature for 15 minutes. Fluorescence data acquisition was carried out using a BD Accuri™ C6 Plus Flow Cytometer (BD Biosciences, Franklin Lakes, NJ, USA; RRID: SCR_019591), with a minimum of 10,000 events collected per sample. PI-positive cells were quantified using the FL2 channel (excitation 488 nm, emission 585/40 nm). Data analysis was performed using FCS Express Flow Cytometry Analysis Software (*De Novo* Software, Pasadena, CA, USA; RRID: SCR_016431). Each experimental procedure was executed in triplicate and independently replicated at least twice. The results were expressed as the percentage of PI-positive cells relative to the entire cellular population.

### Statistical analysis

All data are presented as the mean ± standard deviation (SD) derived from at least three independent experiments, each performed in triplicate. The normality of the input data was tested with the Shapiro-Wilk normality test. For comparisons between multiple groups, statistical significance was assessed using one-way ANOVA, followed by the Tukey *post hoc* test. A p-value < 0.05 was considered statistically significant. Exact p-values are provided where applicable, and significance levels are indicated in the corresponding figure legends. All statistical analyses were performed using IBM SPSS Statistics v.22 (IBM Corp., Armonk, NY, USA; RRID: SCR_002865), and graphical representations were generated using GraphPad Prism v.8 (GraphPad Software, San Diego, CA, USA; RRID: SCR_002798).

## Results

### Physicochemical characteristics of the peptide sequences

The physicochemical properties of the peptide sequences, calculated using the Peptide Property Calculator v3.1, are summarized in **Table 1**. All analyzed peptides exhibited a net positive charge, a feature commonly associated with interactions with negatively charged cellular membranes. Regarding hydrophobicity, only the PC peptide contained a measurable proportion of hydrophobic residues (18.52%). However, the overall amino acid composition of all peptides, characterized by a predominance of charged residues, supports their solubility under aqueous conditions. Both CIB1R and PS sequences are enriched in basic amino acids, which may contribute to electrostatic interactions with anionic membrane components. No visible precipitation was observed for any of the peptides under the experimental conditions, indicating adequate stability in solution.

**Table 1:** Physicochemical characteristics of the peptide sequences.

Peptides	Sequence Composition	# Amino Acids	Basic (%)	Neutral (%)	Acids (%)	Hydrophobic (%)	Net charge	Isoelectric point	Molecular weight
CIB1R	H3N+-PKPKKKQ-COO-	7	57.14	42.86	–	–	+4	11.11	853 Da
PS	H3N+-KQKPKKP-COO-	7	57.14	42.86	–	–	+4	11.11	853 Da
PC	H3N+-GKSESQMDITDINTPKPKKKQRWTPLE-COO-	27	22.22	44.44	14.81	18.52	+2	10.12	3.15 kDa
CTT	H3N+-CTTHWGFTLC-COO-	10	10	40	–	50	0	6.93	1.16 kDa

CIB1R, Peptide derived from the amino-terminal end of the neprilysin protein; PS, Peptide characterized by a disordered sequence; PC, Peptide derived from the cytoplasmic domain; H3N+, Amino-terminal end; COO-, Carboxyl-terminal end; Da, Dalton; KDa, Kilodalton.

### Effect of CIB1R peptide on cell viability

**Figure 1:**
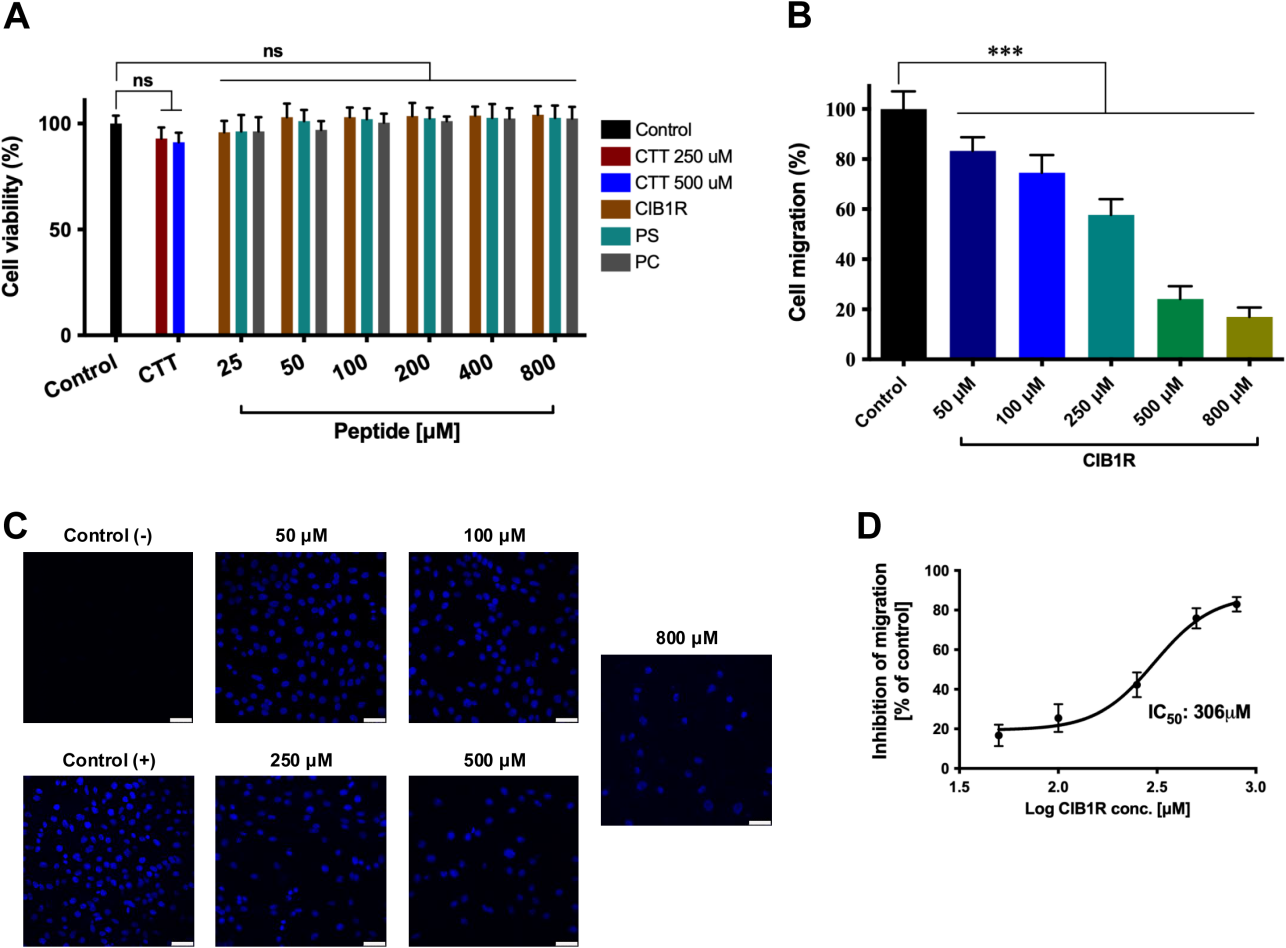
Effects of CIB1R, PS, PC, and CTT peptides on cell viability and dose-response curve of CIB1R for migration in A549 cells. **(A)** Cell viability was assessed at 24 hours using the Alamar Blue assay across the indicated concentrations. No significant differences were observed compared to the control. **(B)** Evaluation of cell migration in response to increasing concentrations of CIB1R peptide. **(C)** Quantitative analysis of cell migration after 24 hours of treatment with CIB1R peptide. **(D)** Dose–response curve showing the inhibition of cell migration by CIB1R, used to determine the IC_50_ value. Data are presented as mean ± SD from three independent experiments. Statistical significance is indicated as follows: *p < 0.05, **p < 0.01, ***p < 0.001 versus control. Scale bars represent 50 µm.

The effect of the CIB1R peptide on A549 cell viability was assessed using the Alamar Blue assay, which reflects cellular metabolic activity. Cells were exposed to peptide concentrations ranging from 25 to 800 µM for 24 hours. Across all tested concentrations, CIB1R did not produce statistically significant changes in cell viability relative to the untreated control. Similar results were observed for the PS and PC peptides, which also did not significantly affect metabolic activity under the same experimental conditions. The control peptide CTT, evaluated at 250 and 500 µM, produced a modest reduction in cell viability (7% and 9%, respectively); however, these differences were not statistically significant. Overall, these findings indicate that, within the experimental conditions tested, none of the peptides induced measurable cytotoxic effects in A549 cells over the 24-hour exposure period. These results support the interpretation that subsequent effects observed in functional assays are unlikely to be attributable to overt loss of cell viability (**Figure 1A**).

### Inhibition of cell migration by CIB1R

**Figure 2:**
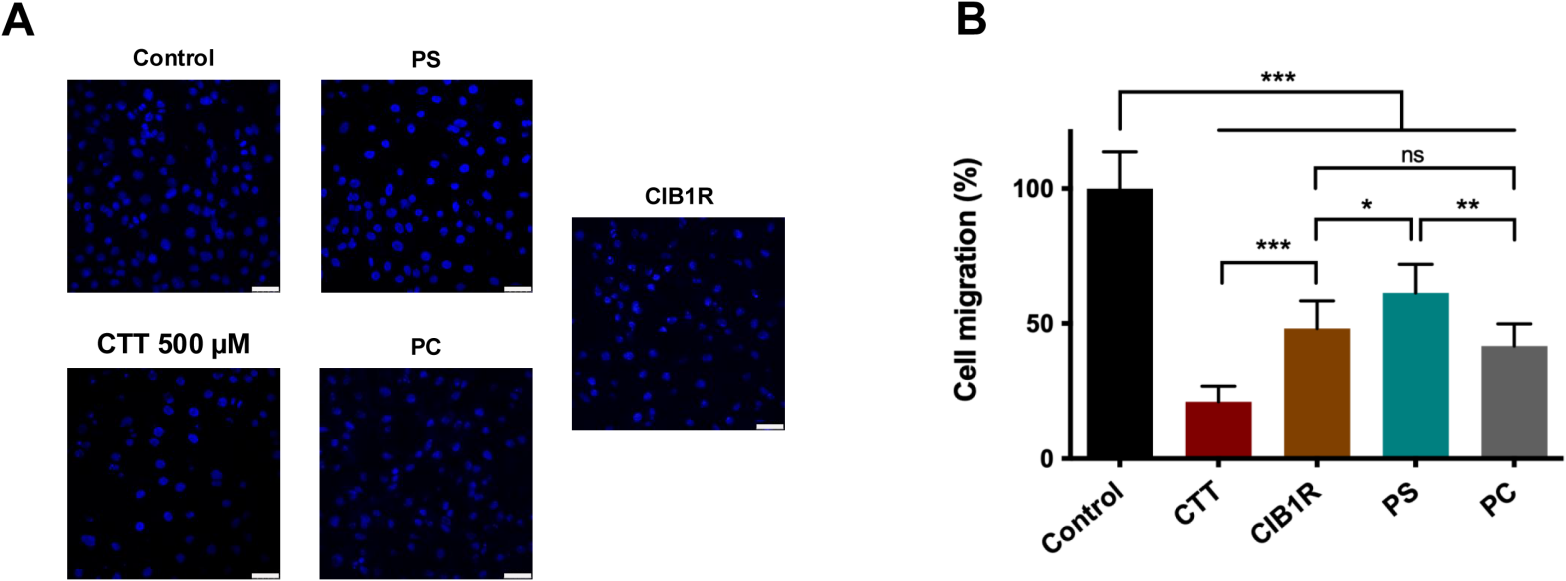
Effects of CIB1R, PS, PC, and CTT peptides on cell migration. **(A)** Representative images showing the effect of peptides on A549 cell migration. **(B)** Quantitative analysis of cell migration at 24 hours in A549 cells treated with each peptide. Data are presented as mean ± SD from three independent experiments. Statistical significance is indicated as follows: *p < 0.05, **p < 0.01, ***p < 0.001. Comparisons were performed against the control group unless otherwise indicated. Scale bars represent 50 µm. ns, no significance.

To evaluate the effect of CIB1R on cell migratory capacity, a Transwell migration assay was performed using concentrations ranging from 50 to 800 µM (**Figure 1B**). Migrated cells were quantified by counting nuclei on the lower surface of the membrane. CIB1R treatment was associated with a concentration-dependent reduction in cell migration relative to the untreated control. As shown in **Figures 1B, C**, migration was reduced by 16.7%, 25.4%, 42.3%, 75.8%, and 82.9% at 50, 100, 250, 500, and 800 µM, respectively, in the presence of a 10% fetal bovine serum gradient. In the absence of a chemoattractant gradient, no migration was detected, confirming the dependence of the assay on chemotactic stimulation. Based on these data, the half-maximal inhibitory concentration (IC_50_) of CIB1R for migration was estimated by nonlinear regression analysis (log[dose] vs. % inhibition), yielding a value of 306 µM (**Figure 1D**). To compare peptide activity under equivalent conditions, the effects of CIB1R, PS, and PC were evaluated at 306 µM and CTT at 500 µM. At this concentration, PS showed 61.4% of cell migration, whereas cells treated with PC showed 41.6%; both were statistically different from the control (**Figures 2A, B**). CIB1R produced approximately 50% inhibition, consistent with its calculated IC_50_. The control peptide CTT (500 µM) reduced migration by 78.9%, consistent with its previously reported inhibitory activity. Notably, the scrambled peptide PS exhibited inhibitory activity comparable to CIB1R under these conditions. This observation suggests that the anti-migratory effects of these peptides may not be exclusively dependent on sequence-specific interactions, but could also be influenced by shared physicochemical properties, such as their high positive charge and potential for electrostatic interactions with cellular components. Collectively, these results demonstrate that basic peptides evaluated in this study reduce A549 cell migration under *in vitro* conditions; however, the extent to which these effects are mediated by sequence-specific mechanisms requires further investigation.

### CIB1R peptide reduces cell invasion

**Figure 3:**
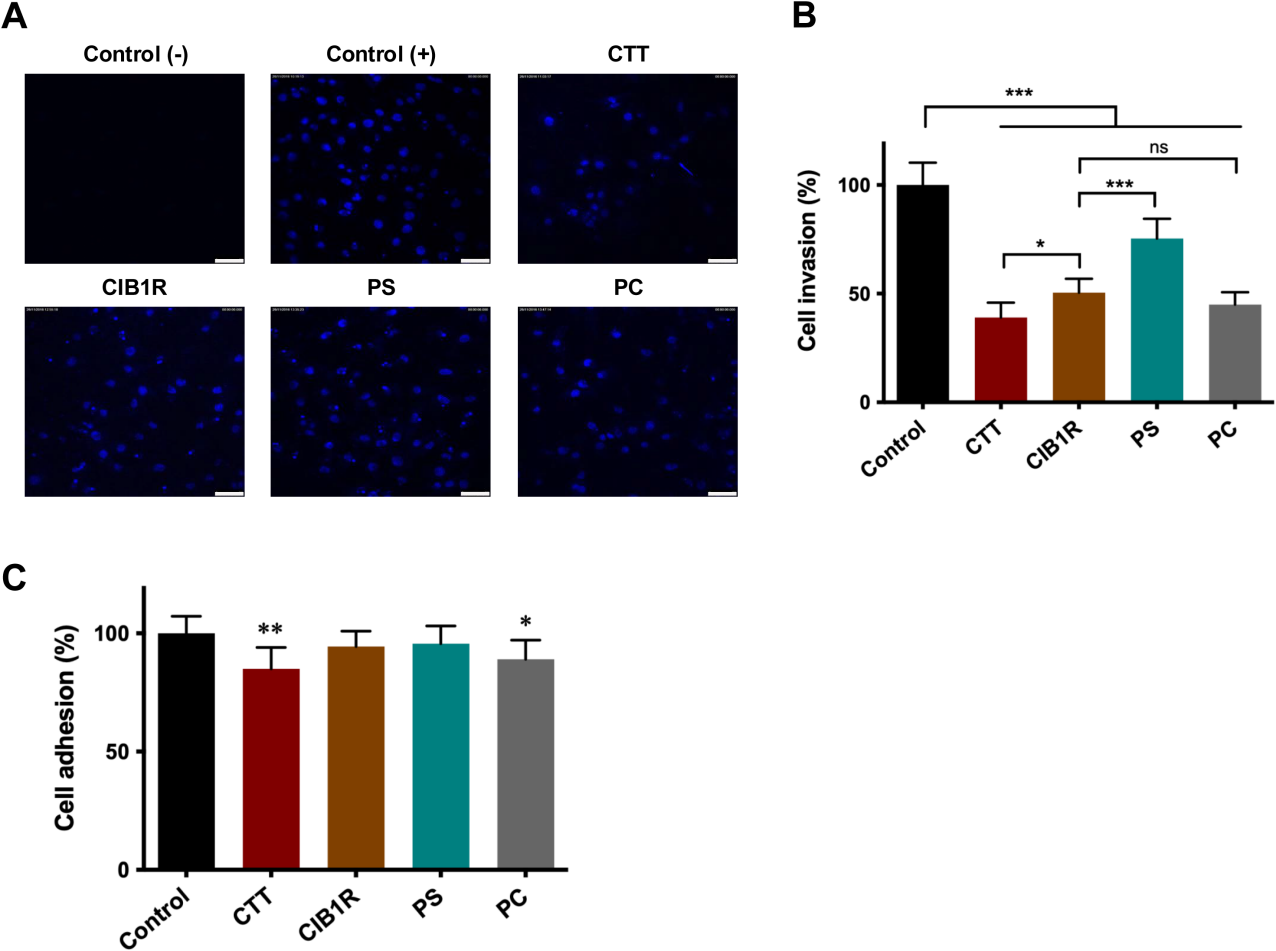
Effects of CIB1R, CTT, PS, and PC peptides on cell invasion and adhesion. **(A)** Representative images showing the effect of CIB1R, CTT, PS, and PC peptides on cell invasion after 24 hours. **(B)** Quantitative analysis of cell invasion in A549 cells treated with the indicated peptides. **(C)** Assessment of cell adhesion to the extracellular matrix after 1 hour of peptide treatment. Data are presented as mean ± SD from three independent experiments. Statistical significance is indicated as follows: *p < 0.05, **p < 0.01, ***p < 0.001. Comparisons were performed against the control group unless otherwise indicated. Scale bars represent 50 µm. ns, no significance.

A concentration of 0.6 mg/mL Matrigel combined with 10% FBS produced a homogeneous and reproducible invasion pattern. The effects of CIB1R, PS, PC (306 µM), and CTT (500 µM) on cell invasion were assessed after 24 hours. All peptides reduced the number of invading cells relative to the control (**Figures 3A, B**). CIB1R and PC produced moderate invasion (~50% and 44.9%, respectively), whereas CTT showed a stronger inhibitory effect (~39% residual invasion). Notably, the scrambled peptide PS exhibited a weaker reduction of invasion (~25%), in comparison with the effect observed for CIB1R under the same conditions. These findings indicate that the inhibitory effects on invasion are not strictly dependent on peptide sequence and may instead reflect shared physicochemical characteristics among the peptides, such as their cationic nature. Taken together, these results demonstrate that all peptides evaluated in this study can reduce A549 cell invasion *in vitro*; however, the underlying mechanisms and the contribution of sequence-specific interactions remain to be determined.

### Inhibition of cell adhesion

Cell adhesion was evaluated using Matrigel-coated plates, with adhesion quantified through Alamar Blue reduction relative to untreated controls (set as 100%). At the tested concentrations (306 µM for CIB1R, PS, and PC; 500 µM for CTT), all peptides produced modest reductions in cell adhesion after 1 hour of incubation. CIB1R reduced adhesion by 5.5%, while PS, PC, and CTT reduced adhesion by 4.3%, 10.9%, and 14.9%, respectively. Statistically significant differences relative to the control were observed for PC and CTT (**Figure 3C**). Overall, the effects on adhesion were limited in magnitude compared to those observed in migration and invasion assays, suggesting that reduced adhesion alone does not fully account for the observed inhibition of cell motility.

### Analysis of peptide-associated fluorescence by flow cytometry

**Figure 4:**
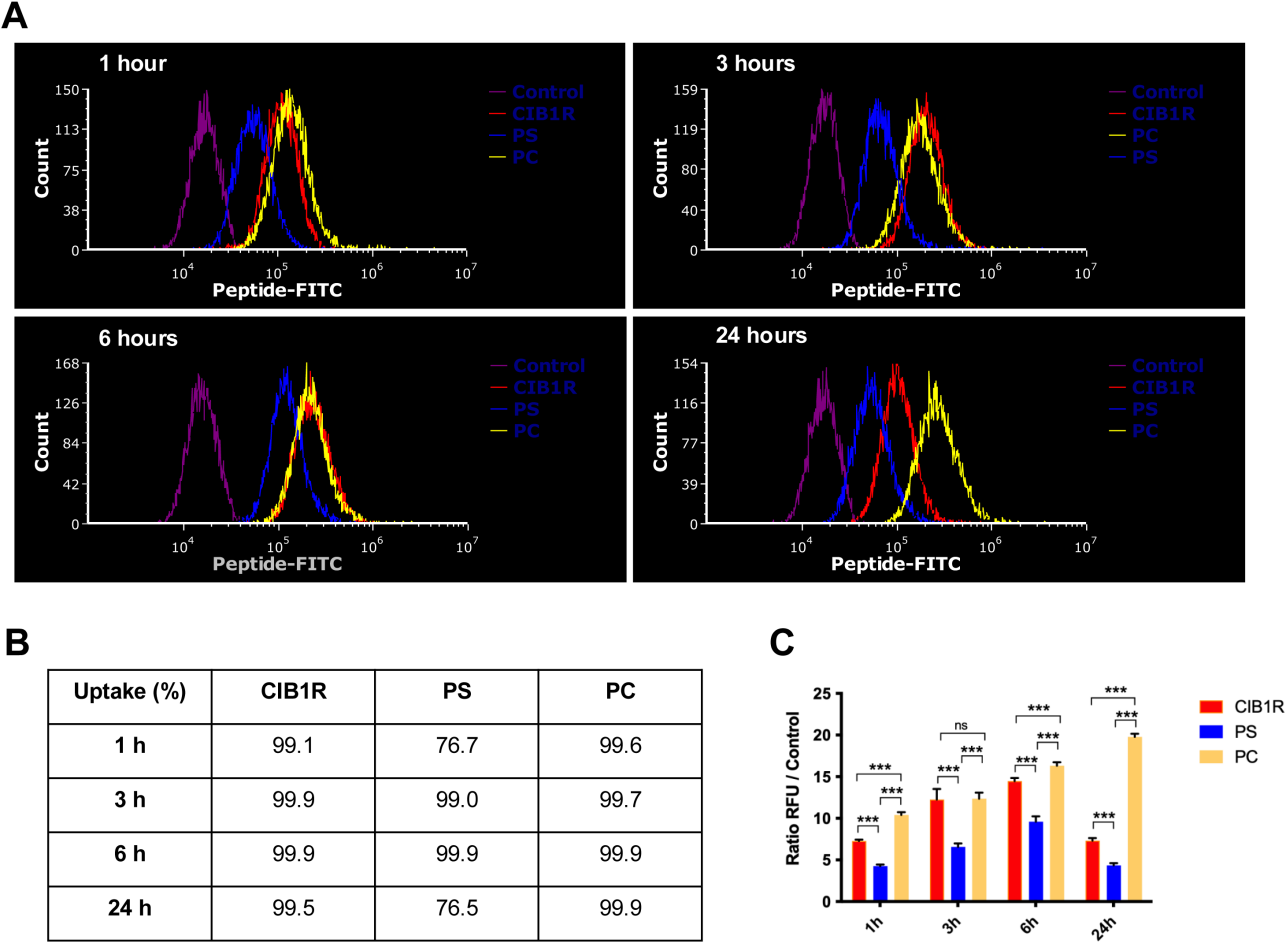
Peptide-associated fluorescence in A549 cells analyzed by flow cytometry. **(A)** Representative histograms showing fluorescence signals in A549 cells after incubation with FITC-labeled peptides at 1, 3, 6, and 24 hours (37 °C). Data were analyzed using FCS Express (*De Novo* Software). **(B)** Percentage of fluorescence-positive cells at each time point for the indicated peptides. **(C)** Relative fluorescence intensity (RFU) of peptide-associated signal in A549 cells, expressed as the ratio relative to control cells. Measurements were obtained using an Accuri C6 flow cytometer. Data are presented as mean ± SD from three independent experiments. Statistical significance is indicated as follows: *p < 0.05, **p < 0.01, ***p < 0.001. Comparisons were performed against the control group unless otherwise indicated. ns, no significance.

The association of FITC-labeled peptides (CIB1R, PS, and PC) with A549 cells was evaluated by flow cytometry at 1, 3, 6, and 24 hours using a concentration of 306 µM. A high percentage of cells exhibited fluorescence signals under all treatment conditions. Both CIB1R and PC were associated with fluorescence in approximately 99% of analyzed cells across all time points, whereas PS showed a lower proportion at early and late time points (~76%), reaching similar levels at intermediate times (3–6 hours) (**Figures 4A, B**). Quantitative analysis of fluorescence intensity (relative fluorescence units, RFU) revealed distinct temporal patterns among the peptides (**Figure 4C**). PS exhibited a transient increase in fluorescence, peaking at 6 hours and decreasing thereafter. CIB1R displayed a similar but more pronounced pattern, with peak fluorescence at 6 hours followed by partial reduction at 24 hours. In contrast, PC showed a progressive increase in fluorescence intensity over time. These data indicate differential temporal dynamics in peptide-associated fluorescence; however, they do not distinguish between membrane-associated and intracellular fluorescence signals.

### Confocal microscopy analysis of peptide-associated fluorescence

**Figure 5:**
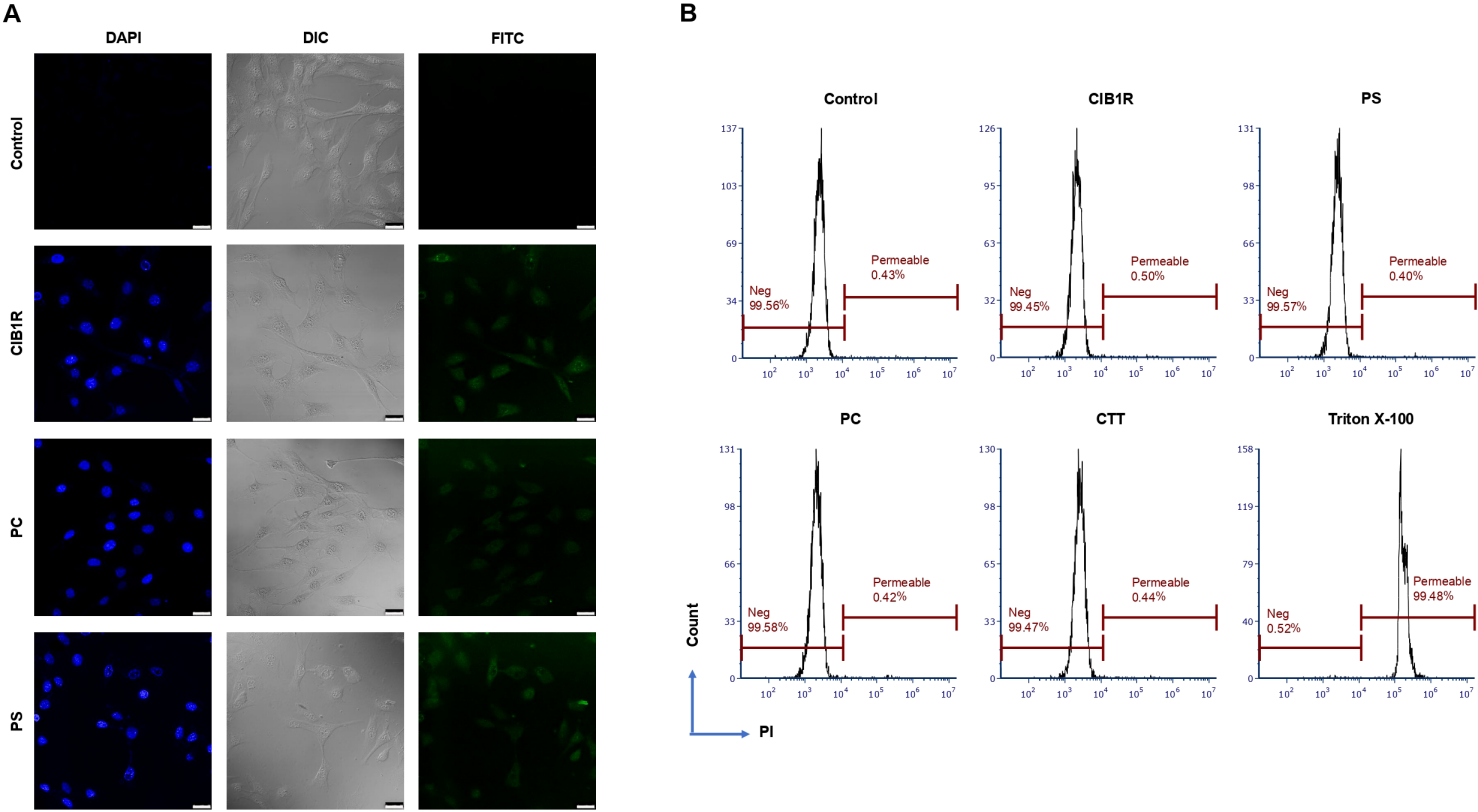
Peptide-associated fluorescence and cell membrane integrity assessment in A549 cells. **(A)** Representative confocal microscopy images of A549 cells after 6 hours of incubation with FITC-labeled CIB1R, PS, and PC peptides (306 µM, at 37 °C). The fluorescence signal is shown as a peptide-associated signal. **(B)** Representative histograms of propidium iodide **(PI)** uptake in A549 cells treated with CIB1R, PS, PC and CTT peptides. No significant increase in PI-positive cells was observed under the experimental conditions. For each sample, 10,000 events were analyzed using an Accuri C6 flow cytometer, and data were processed with FCS Express (*De Novo* Software). Scale bars represent 25 µm.

Confocal microscopy was used to qualitatively assess the spatial distribution of FITC-associated fluorescence in A549 cells following 6 hours of incubation with peptides (306 µM) (**Figure 5A**). Fluorescence signals were observed in peptide-treated cells and appeared diffusely distributed throughout the cellular area. Importantly, the experimental design did not include Z-stack acquisition or orthogonal reconstruction, limiting the ability to assess subcellular distribution in three dimensions. Therefore, the observed fluorescence should be interpreted as peptide-associated signal rather than definitive evidence of intracellular or nuclear localization.

### Assessment of cell membrane integrity

Cell membrane integrity was evaluated by measuring propidium iodide (PI) uptake using flow cytometry. A549 cells were treated with CIB1R, PS, PC, and CTT peptides (306 µM) for 24 hours under standard culture conditions. As shown in **Figure 5B**, fluorescence intensity histograms revealed no appreciable increase in PI-positive cells following peptide treatment compared with untreated controls. In all cases, the proportion of PI-positive cells remained below 1%, indicating no detectable loss of plasma membrane integrity under the conditions tested. In contrast, treatment with Triton X-100 (5 minutes) resulted in a marked increase in PI uptake, with over 99% of cells exhibiting high fluorescence intensity, confirming effective membrane permeabilization and validating the assay. These results indicate that, within the experimental conditions evaluated, peptide treatment was not associated with detectable membrane disruption as assessed by PI exclusion. However, given the differences in experimental context between PI-based permeability assays and fluorescence microscopy analyses, these findings should be interpreted independently. Overall, the data suggest that peptide exposure does not induce overt membrane damage detectable by PI uptake, although additional approaches would be required to fully characterize potential effects on membrane dynamics or permeability.

## Discussion

In this study, we evaluated the effects of the CIB1R peptide on cellular processes associated with tumor progression, including migration, invasion, and adhesion, using an *in vitro* model of A549 non-small cell lung cancer (NSCLC) cells. Our results show that CIB1R reduces cell migration and invasion under controlled experimental conditions, without inducing significant changes in cell viability during short-term exposure. CIB1R is derived from a basic region of the cytoplasmic domain of neprilysin (NEP), a membrane-associated metalloprotease that has been implicated in the regulation of cell migration and intracellular signaling pathways in several cancer types (11, 13). However, in the present study, NEP expression was not experimentally validated in A549 cells. Therefore, any mechanistic interpretation linking the observed effects of CIB1R to NEP-associated signaling pathways should be considered hypothetical and based on prior reports rather than direct evidence from this model system.

Fluorescence-based analyses demonstrated a strong peptide-associated signal in treated cells. Nevertheless, the available data do not allow definitive discrimination between membrane-associated fluorescence and true intracellular localization. In particular, the absence of three-dimensional imaging approaches, such as Z-stack acquisition or orthogonal reconstruction, and the lack of quantitative colocalization analysis limit the interpretation of subcellular distribution. Consequently, the observed fluorescence should be interpreted as peptide-associated signal rather than conclusive evidence of cytoplasmic or nuclear localization. Although previous studies have reported that cationic peptides can enter cells via endocytosis or direct translocation mechanisms (14, 15), the current data do not permit identification of the uptake pathway for CIB1R or PC. Furthermore, flow cytometry measurements of fluorescence positivity do not distinguish between peptides bound to the cell surface and those internalized. Therefore, the mechanisms underlying peptide–cell association remain unresolved and require further investigation using validated internalization assays. Importantly, no increase in membrane permeability was detected using propidium iodide (PI) exclusion assays. However, given the methodological differences between PI-based permeability measurements and fluorescence microscopy analyses, these findings should be interpreted independently. While the results suggest that peptide exposure does not induce overt membrane disruption under the conditions tested, they do not exclude the possibility of surface association or alternative modes of interaction with the plasma membrane.

Previous studies have reported reduced NEP expression in NSCLC and in A549 cells, as well as in other malignancies such as prostate and breast cancer, where it has been associated with tumor progression (10, 16). Most investigations have focused on correlations between NEP expression levels and clinical parameters, including tumor grade, metastatic status, and patient survival (17, 18). Although experimental approaches involving NEP overexpression have provided insights into its role in regulating cell migration, there are currently no reports evaluating short peptide sequences derived from the cytoplasmic domain of NEP as modulators of tumor cell behavior. In this context, our findings provide initial functional evidence that peptides derived from this region can influence cell migration and invasion *in vitro*. However, it is important to note that these effects are observed under controlled experimental conditions and at relatively high peptide concentrations (19).

Previous studies have extensively investigated the role of neprilysin (NEP) in the regulation of cell migration across multiple cancer models. For instance, expression of a catalytically inactive NEP variant has been shown to reduce migration by approximately 54% in PC3 prostate cancer cells (13). Similarly, NEP overexpression in SKOV3 ovarian carcinoma cells resulted in a reduction in migration of approximately 62% (20), an effect that has been partially attributed to the combined contribution of its cytoplasmic and extracellular domains. In particular, the extracellular domain of NEP is capable of degrading migration-promoting peptides such as endothelin-1 (ET-1), thereby modulating downstream signaling pathways including focal adhesion kinase (FAK) activation (21).

In breast cancer models, restoration of NEP expression in MCF-7 and MDA-MB-231 cells has also been associated with reductions of approximately 50% in migration and invasion (22), supporting the concept that NEP functions as a negative regulator of tumor cell invasiveness. Collectively, these studies highlight the relevance of NEP in controlling cell motility through both enzymatic and non-enzymatic mechanisms. In the present study, treatment with CIB1R and PC peptides resulted in moderate inhibition of migration and invasion in A549 cells, with reductions of approximately 50% under the experimental conditions tested. While these values fall within a similar range to those reported for NEP-based modulation, direct comparisons should be interpreted with caution. In contrast to genetic overexpression or full-length protein models, short peptides such as CIB1R are subject to distinct pharmacodynamic and biochemical constraints, including potential proteolytic degradation, limited stability, and the requirement to reach effective intracellular or membrane-associated concentrations (23, 24).

Importantly, the observation that the scrambled peptide PS exhibited comparable inhibitory effects to those of CIB1R indicates that the biological activity observed in this study may not be exclusively dependent on sequence-specific interactions derived from the NEP cytoplasmic domain. Instead, these findings suggest that shared physicochemical properties, particularly the high positive charge and amino acid composition of these peptides, may contribute to their effects on cell migration and invasion. Consistent with this interpretation, cationic peptides are known to interact with negatively charged components of the plasma membrane and extracellular matrix, potentially influencing cell adhesion and motility through electrostatic mechanisms. Therefore, while the origin of CIB1R from the NEP cytoplasmic domain provides a biologically relevant framework, the current data do not allow definitive attribution of its effects to specific NEP-mediated signaling pathways.

Previous studies have proposed that NEP may regulate cell migration through interactions involving its cytoplasmic domain and cytoskeletal-associated proteins, including members of the ERM (ezrin/radixin/moesin) complex (25–27). These proteins function as molecular linkers between the actin cytoskeleton and transmembrane receptors such as CD44, CD43, and ICAM family members, thereby modulating processes including adhesion, motility, and cellular morphology. However, in the absence of direct biochemical or molecular evidence in the present study, the involvement of these pathways should be considered speculative. Taken together, our findings suggest that short cationic peptides derived from, or inspired by, the cytoplasmic domain of NEP can modulate cell migration and invasion *in vitro*. However, the relative contribution of sequence-specific interactions *versus* general physicochemical effects remains to be determined.

In addition, electrostatic interactions involving phospholipids such as phosphatidylinositol 4,5-bisphosphate (PIP2) have been described in related systems, where basic residues mediate membrane-associated signaling events (28).

In this context, it is plausible that the peptides evaluated in this study may influence cell behavior through interactions with membrane-associated or cytoskeletal regulatory components. However, given the absence of direct biochemical or molecular evidence, the involvement of specific pathways such as ERM-mediated interactions, PIP2 binding, or modulation of signaling molecules including PTEN should be interpreted as hypothetical rather than definitive. Notably, alterations in NEP expression have also been described in endothelial cells within the tumor microenvironment, where they have been associated with vascular remodeling and metastatic dissemination (29). This broader biological context supports the relevance of NEP-related pathways in cancer progression, although their direct relationship with the effects observed for the peptides in this study remains to be established. Furthermore, the present findings are consistent with previous reports describing bioactive peptides derived from pathological fluids, such as ascitic or pleural effusions, which have been shown to modulate tumor-related processes including cell migration, invasion, apoptosis, and angiogenesis (30). In this regard, peptides derived from functional domains of larger proteins may represent a relevant source of biologically active molecules capable of influencing tumor cell behavior.

### Strengths of the study

This study presents several strengths that support its relevance as an initial functional evaluation of NEP-derived peptides in cancer biology. First, it introduces an exploratory approach by assessing the effects of short peptides derived from the cytoplasmic domain of neprilysin (NEP) on cellular processes associated with tumor progression, including migration, invasion, and adhesion. To our knowledge, the use of peptides derived from this specific domain as modulators of tumor cell behavior has not been previously reported, thereby providing a novel experimental framework.

A key strength of the study lies in the use of multiple complementary functional assays to evaluate cell migration, invasion, and adhesion. These approaches, including Transwell-based systems and metabolic quantification methods, are widely established and provide reproducible and quantitative measurements under controlled *in vitro* conditions. The consistency of the observed inhibitory effects across independent assays supports the robustness of the functional findings.

Additionally, the study demonstrates that peptide treatment reduces migration and invasion without significantly affecting cell viability during short-term exposure. This distinction is relevant, as it indicates that the observed effects are not attributable to overt cytotoxicity, but rather to modulation of specific cellular behaviors associated with tumor progression.

Another strength is the inclusion of multiple peptide sequences with related physicochemical properties, including CIB1R, PC, and the scrambled control PS, as well as the reference peptide CTT. This comparative design allows for a more rigorous interpretation of the results by highlighting both shared and differential effects among peptides. In particular, the observation that peptides with similar charge and composition exhibit comparable biological activity provides important insight into the contribution of physicochemical properties to the observed effects.

Furthermore, the use of the A549 cell line, a well-characterized model of non-small cell lung cancer (NSCLC), enables direct comparison with existing literature and supports the relevance of the findings within a widely studied experimental system.

Finally, the study establishes a functional foundation for investigating peptide-based modulation of tumor cell behavior. By integrating experimental observations with existing knowledge on NEP-related pathways, this work contributes to the conceptual development of peptide-based strategies for studying mechanisms involved in cancer cell motility.

### Limitations of the study

Several limitations of this study should be considered when interpreting the results.

First, the experimental findings were obtained exclusively using a single *in vitro* model (A549 cells), which does not capture the biological heterogeneity of lung cancer. While this cell line represents a well-characterized model of non-small cell lung cancer, the extent to which the observed effects are generalizable across different genetic and phenotypic backgrounds remains uncertain. Second, all experiments were conducted under *in vitro* conditions, which do not recapitulate the complexity of the tumor microenvironment. Factors such as stromal interactions, immune components, oxygen gradients, and soluble mediators are absent in this system and may significantly influence tumor cell behavior and treatment responses.

Third, the study focused on functional assays evaluating migration, invasion, and adhesion, without directly investigating the underlying molecular mechanisms. Although mechanistic hypotheses were discussed based on previous literature, no biochemical or molecular validation, such as protein interaction assays or pathway-specific analyses, was performed. Consequently, the mechanisms responsible for the observed effects remain unresolved.

An additional limitation relates to the interpretation of peptide-associated fluorescence. The confocal microscopy data do not allow a clear distinction between membrane-associated and internalized peptide, and the absence of three-dimensional imaging or quantitative colocalization analysis limits conclusions regarding subcellular localization. Furthermore, the experimental conditions used for DAPI staining and propidium iodide assays differ, preventing direct comparison of membrane permeability and intracellular localization outcomes.

The lack of direct assessment of endogenous NEP expression in A549 cells represents another important limitation. As a result, mechanistic interpretations involving NEP-related pathways cannot be experimentally substantiated within the current study and should be interpreted with caution. Additionally, all functional assays were performed under serum-free conditions. While this approach allows controlled evaluation of cell migration and invasion, it eliminates peptide degradation mediated by serum proteases and may therefore overestimate peptide stability and apparent biological activity. In this context, the reported IC_50_ value (306 µM) should be interpreted within the constraints of the experimental system rather than as an indicator of potency under physiological conditions. The relatively high concentrations required to achieve biological effects further raise the possibility that the observed activity may be influenced, at least in part, by non-specific physicochemical interactions rather than sequence-specific mechanisms. Finally, the study was designed to assess short-term effects (up to 24 hours), which does not allow evaluation of long-term cellular responses, including adaptive mechanisms, resistance development, or sustained phenotypic changes.

### Future directions

Building on the findings of the present study, several research directions emerge as priorities for further investigation. A key next step is the evaluation of peptide activity in more complex biological systems. In particular, validation in additional cancer cell lines representing diverse genetic and phenotypic backgrounds will be essential to determine the generalizability of the observed effects. Complementary studies in preclinical models will further enable the assessment of peptide behavior in physiologically relevant environments, including their impact on tumor progression, metastatic dissemination, and tissue-specific responses. Another important area of investigation involves the elucidation of the molecular mechanisms underlying the observed effects. Future studies should aim to validate potential peptide–target interactions using biochemical and molecular approaches, including protein interaction assays, pathway-specific analyses, and functional modulation studies. These efforts will be critical to distinguish between sequence-specific mechanisms and broader physicochemical effects. In parallel, it will be necessary to characterize the pharmacological properties of these peptides under conditions that more closely resemble the physiological environment. This includes evaluating peptide stability in the presence of serum, susceptibility to proteolytic degradation, and the influence of these factors on biological activity and effective concentration ranges. Given the fluorescence-based observations reported in this study, further work is also required to clarify the mechanisms of peptide–cell association. Approaches such as high-resolution imaging, three-dimensional reconstruction, and quantitative subcellular analysis will be important to determine whether the peptides are internalized or primarily associated with the cell surface.

In addition, expanding the analysis to include other cellular systems relevant to tumor progression, such as endothelial cells involved in angiogenesis, may provide further insight into the broader biological activity of these peptides. Finally, the identification and optimization of peptide properties, including sequence modifications or delivery strategies, may improve their stability, specificity, and functional potency. Such efforts could support the development of peptide-based tools for investigating mechanisms of cell motility and, potentially, their future translation into more advanced preclinical applications.

## Conclusion

In this study, the CIB1R peptide reduced cell migration and invasion in A549 lung carcinoma cells under controlled *in vitro* conditions, without significantly affecting cell viability during short-term exposure. These findings indicate that the observed effects are not attributable to overt cytotoxicity, but rather to modulation of cellular behaviors associated with tumor progression. Comparative analyses with structurally related peptides revealed that the inhibitory effects were not exclusive to CIB1R. In particular, the scrambled peptide PS exhibited comparable activity, indicating that the observed biological effects are not strictly dependent on sequence-specific interactions. Instead, these results suggest that shared physicochemical properties such as the cationic nature of the peptides may contribute to their influence on cell migration and invasion. Although fluorescence-based approaches demonstrated strong peptide-associated signals in treated cells, the current experimental design does not allow definitive discrimination between membrane-associated and intracellular localization. Therefore, conclusions regarding peptide internalization, subcellular distribution, or nuclear localization cannot be established based on the available data. Similarly, although no increase in membrane permeability was detected by propidium iodide exclusion assays, these findings should be interpreted in the specific experimental context and do not exclude alternative modes of peptide–cell interaction. Overall, this study provides initial functional evidence that short cationic peptides derived from, or inspired by, the cytoplasmic domain of neprilysin can modulate cellular processes associated with tumor progression *in vitro*. However, the underlying mechanisms, the specificity of interaction, and the relevance of these effects under physiological conditions remain to be determined. 

## Data Availability

The original contributions presented in the study are included in the article/**Supplementary Material**. Further inquiries can be directed to the corresponding authors.
