# Supplementary material for: Evaluation of the CIB1R peptide derived from the cytoplasmic domain of neprilysin on cell migration in an in vitro model of lung cancer
Source: Front Oncol. 2026 Jun 23;16:1825515. doi: 10.3389/fonc.2026.1825515 (PMC13337379; doi:10.3389/fonc.2026.1825515)
Supplement: Supplementary file 1 [file Table1.docx]

**Table S1.** Analytical characterization and purity of peptide sequences by HPLC and mas spectrometry.

| **Peptide** | **Purity (%)** | **Theoretical Mass (Da)** | **Ionization Mode** | **QC Method** |
| --- | --- | --- | --- | --- |
| CIB1R | ≥95 | 853.0 | ESI (+) | RP-HPLC + ESI-MS |
| PS | ≥95 | 853.0 | ESI (+) | RP-HPLC + ESI-MS |
| PC | ≥95 | 3150.0 | ESI (+) | RP-HPLC + ESI-MS |
| CTT | ≥95 | 1160.0 | ESI (+) | RP-HPLC + ESI-MS |
